# Risk of postoperative major adverse cerebrovascular events in patients with spontaneous intracranial hematoma stratified by type 2 diabetes mellitus

**DOI:** 10.3389/fnhum.2025.1654763

**Published:** 2025-09-11

**Authors:** Junhua Yang, Sihui Wang, Xiangtian Ji, Yu Sun, Jingyu Feng, Bin Liu, Jun Yang

**Affiliations:** ^1^Department of Neurosurgery, Peking University Third Hospital, Beijing, China; ^2^Center for Precision Neurosurgery and Oncology of Peking University Health Science Center, Beijing, China

**Keywords:** spontaneous intracranial hematoma, diabetes mellitus, surgery, complications, stroke

## Abstract

**Background and purpose:**

Diabetes Mellitus (DM) is a common concomitant disease of spontaneous intracranial hemorrhage (ICH). Postoperative major adverse cerebrovascular events (post-MACEs) may diminish the surgical benefits of patients with ICH. However, evidence regarding the impact of DM on post-MACEs remains limited.

**Methods:**

This was a multicenter cohort study that enrolled ICH patients presenting to eight neurosurgery departments between January 1, 2015, and May 31, 2021. Patients were categorized into DM group and no diabetes mellitus (nDM) group, based on the presence or absence of DM. Intergroup comparisons were performed using chi-square tests for categorical variables and Mann–Whitney U tests for continuous variables. Multivariate logistic regression analysis was conducted to assess the impact of DM on post-MACEs and 30-days mortality after adjusting for confounding factors. A stratified analysis was also conducted based on the type of post-MACEs.

**Results:**

A total of 688 ICH patients were included in the study, of whom 576 (83.7%) were classified into the nDM group and 112 (16.3%) into the DM group. Compared with the nDM group, the DM group exhibited significantly higher incidences of overall post-MACEs (28.6%), ischemic post-MACEs (14.3%), hemorrhagic post-MACEs (23.2%), and 30-days mortality (9.8%). After adjusting for potential confounding factors including sex, age, alcohol, coronary heart disease, dyslipidemia, antiplatelet therapy, and intraventricular hemorrhage, DM remained a significant predictor of overall post-MACEs (OR: 1.790, 95%CI: 1.072–2.990, *p* = 0.026), ischemic post-MACEs (OR: 2.139, 95%CI: 1.090–4.197, *p* = 0.027), hemorrhagic post-MACEs (OR: 1.778, 95%CI: 1.015–3.114, *p* = 0.044), and 30-days mortality (OR: 3.593, 95%CI: 1.536–8.406, *p* = 0.003).

**Conclusion:**

In conclusion, this study demonstrates that DM serves as a significant risk factor for ischemic post-MACEs, hemorrhagic post-MACEs, and 30-days mortality among patients with ICH.

## 1 Introduction

Spontaneous intracranial hemorrhage (ICH) is defined as a condition resulting from the rupture of small intracranial blood vessels due to non-traumatic causes (Qureshi et al., 2009; Wilkinson et al., 2018). It ranks as the second most common type of stroke; however, its mortality and disability rates exceed those of ischemic stroke. According to the Global Burden of Disease Study, the incidence of ICH in China is approximately 45/100,000 (GBD 2019 Stroke Collaborators, 2021). Reports indicate that by 2030, the incidence of cerebrovascular disease events in China will increase by approximately 50% compared with 2010 (Tu et al., 2023), thereby imposing a substantial burden on society and families.

Pharmacological and surgical interventions both serve as effective components of comprehensive treatment for ICH (Wu et al., 2020; Zhou et al., 2011). However, for patients with severe ICH, surgery may represent a more favorable option. Clinical studies have demonstrated that rapid removal of the intracranial hematoma can significantly enhance the prognosis in patients with severe ICH (Wu et al., 2020; Zhou et al., 2011; Shi et al., 2019). However, the occurrence of postoperative major adverse cerebrovascular events (post-MACEs) can markedly diminish the surgical benefits for patients with ICH (Shen et al., 2021; Wu et al., 2017; Ren et al., 2018). Therefore, effectively reducing the occurrence of post-MACEs constitutes a critical strategy to improve surgical outcomes.

It has been reported that approximately 14% of ICH patients are comorbid with diabetes mellitus (DM) (Tu et al., 2023). Pathological studies have indicated that small vessel injury represents a critical mechanism by which DM affects multiple organs throughout the body (Antar et al., 2023). Preclinical investigations into ICH have further demonstrated that DM can influence intracranial edema and bleeding volume by altering cerebral vascularization (Antar et al., 2023; Bahadar and Shah, 2021; Gómez-de Frutos et al., 2024). Based on these findings, we hypothesized that DM may contribute to an increased incidence of post-MACEs in ICH patients via its impact on small intracranial blood vessels. Indeed, an increasing number of clinical studies have shown that DM is associated with poorer prognoses in ICH patients (Antar et al., 2023; Liebkind et al., 2018; Boulanger et al., 2016; Cavallari et al., 2021). However, to date, there remains a paucity of clinical evidence regarding the specific effects of DM on post-MACEs in the ICH population. Therefore, to evaluate the potential influence of DM on post-MACEs in ICH patients, we designed this multicenter cohort study.

This study is a multicenter retrospective study. As this study is a retrospective analysis and poses no risk to participants, the Ethics Committee of Peking University Third Hospital waived the requirement for informed consent.

## 2 Materials and methods

### 2.1 Study population

This study is a multicenter retrospective study. According to the inclusion and exclusion criteria, this study continuously included ICH patients who visited the neurosurgery departments of 8 medical institutions in Beijing and Guangzhou city from January 1, 2015 to May 31, 2021.

### 2.2 Inclusion and exclusion criteria

Inclusion criteria for participants of this study were: 1. over 18 years old and under 90 years old; 2. patients with symptoms of ICH such as sudden headache, functional impairment and consciousness disorder; 3. patients diagnosed with ICH by head computed tomography (CT) within 24 h of the onset of the disease.

Exclusion criteria for participants of this study were: (1) cerebral hemorrhage caused by a clear cause, such as amyloidosis, brain tumors, intracranial aneurysms, cerebral arteriovenous malformations, intracranial cavernous hemangiomas, intracranial dural arteriovenous fistulas, cerebral venous malformations, moyamoya disease, brain trauma, venous sinus thrombosis, cerebral infarction, etc. (2) patients with brainstem hemorrhage; (3) patients with ICH not received surgical treatment within 7 days; (4) patients with primary or secondary coagulation dysfunction; (5) patients with malignant tumors; (6) patients with severe cardiopulmonary dysfunction; (7) patients with severe liver and kidney dysfunction; (8) patients received any anticoagulant therapy before the operation (such as: heparin, warfarin, rivaroxaban.); (9) patients with type 1 DM; (10) patients with missing data.

### 2.3 Surgery

The selection of surgical indications and surgical methods is jointly determined by two senior physicians and one or two junior physicians. The attending physician determined the surgical method based on the patient’s disease, family preferences, and surgical indications, such as microscopic craniotomy for hematoma evacuation, endoscopic surgery for hematoma evacuation or minimally invasive surgery.

Surgical indications: (1) ICH with signs of brain herniation; (2) supratentorial cerebral hemorrhage with a volume greater than 30 mL; (3) infratentorial cerebral hemorrhage with a volume greater than 10 mL, etc. (Wu et al., 2020).

All ICH patients underwent head CT within 24 h and 48 h after admission or surgery, respectively. In cases of changes in the patient’s consciousness or at the discretion of the attending physician, a head CT scan was performed promptly.

### 2.4 Data collection

Clinical information of ICH patients obtained from the medical records, included: (1) demographic characteristics: sex, age, height, weight, etc. (2) vascular risk factors: smoking, alcohol, admission blood pressure, etc. (3) medication history: hypertension, dyslipidemia, DM, chronic coronary heart disease, history of cerebral infarction, history of cerebral hemorrhage, antiplatelet therapy, etc. (4) imaging features: hematoma side, hematoma location, hematoma volume, ventricular hematoma, etc. (5) laboratory tests: platelet count, international normalized ratio, admission blood glucose, etc. (6) functional status: Glasgow Coma Scale (GCS) of patients at admission and 30-days, and 30-days mortality. (7) surgical information and complications: surgical method, blood transfusion, and post-MACEs.

### 2.5 Outcomes

The primary outcome was postoperative major adverse cerebrovascular events (post-MACEs), defined as either or combination of ischemic post-MACEs and hemorrhagic post-MACEs.

The secondary outcome was 30-days mortality, defined as all-cause mortality within 30-days after the onset of ICH.

### 2.6 Definition

Ischemic post-MACEs were defined as postoperative acute cerebral infarction occurring after operation within 30 days of ICH.

Hemorrhagic post-MACEs were defined as follows: the hematoma volume increased by more than 33% at any postoperative follow-up CT compared with the previous postoperative follow-up CT within 30 days after the onset of ICH, for patients received minimally invasive surgical treatment; or any postoperative follow-up CT found new high-density shadow at the original hematoma location within 30 days after the onset of ICH, for patients received other surgical methods; or new high-density shadows was found in the non-surgical area at any postoperative follow-up CT within 30 days after the onset of ICH, for patients received any surgical methods (Wu et al., 2017; Shen et al., 2018).

Hematoma location: the frontal lobe, parietal lobe, occipital lobe and temporal lobe were defined as lobar part; while the basal ganglia, thalamus, internal capsule, external capsule, corpus callosum and subtentorial structures of the cerebellum were defined as the deep part.

Hematoma volume: The hematoma volume in ICH patients was measured by the method of A × B × C/2.

### 2.7 Statistical analysis

All categorical variables were described in terms of quantity and percentage; Continuous variables were described by means (standard deviations) or medians (quartiles) when appropriate. When comparing between groups, categorical variables were statistically analyzed using chi-square tests. Continuous variables were statistically analyzed using the Mann–Whitney U test. Multivariate logistic regression analysis was used to correct confounding factors with *p* < 0.05 when comparing between groups. Chi-square test, Mann–Whitney U test and multivariate logistic regression analysis were analyzed by spss 26.0 software (IBM). The forest map was drawn using graphpad prism 10. Bilateral *p* < 0.05 was considered statistically significant.

## 3 Results

### 3.1 Study population

**Figure 1:**
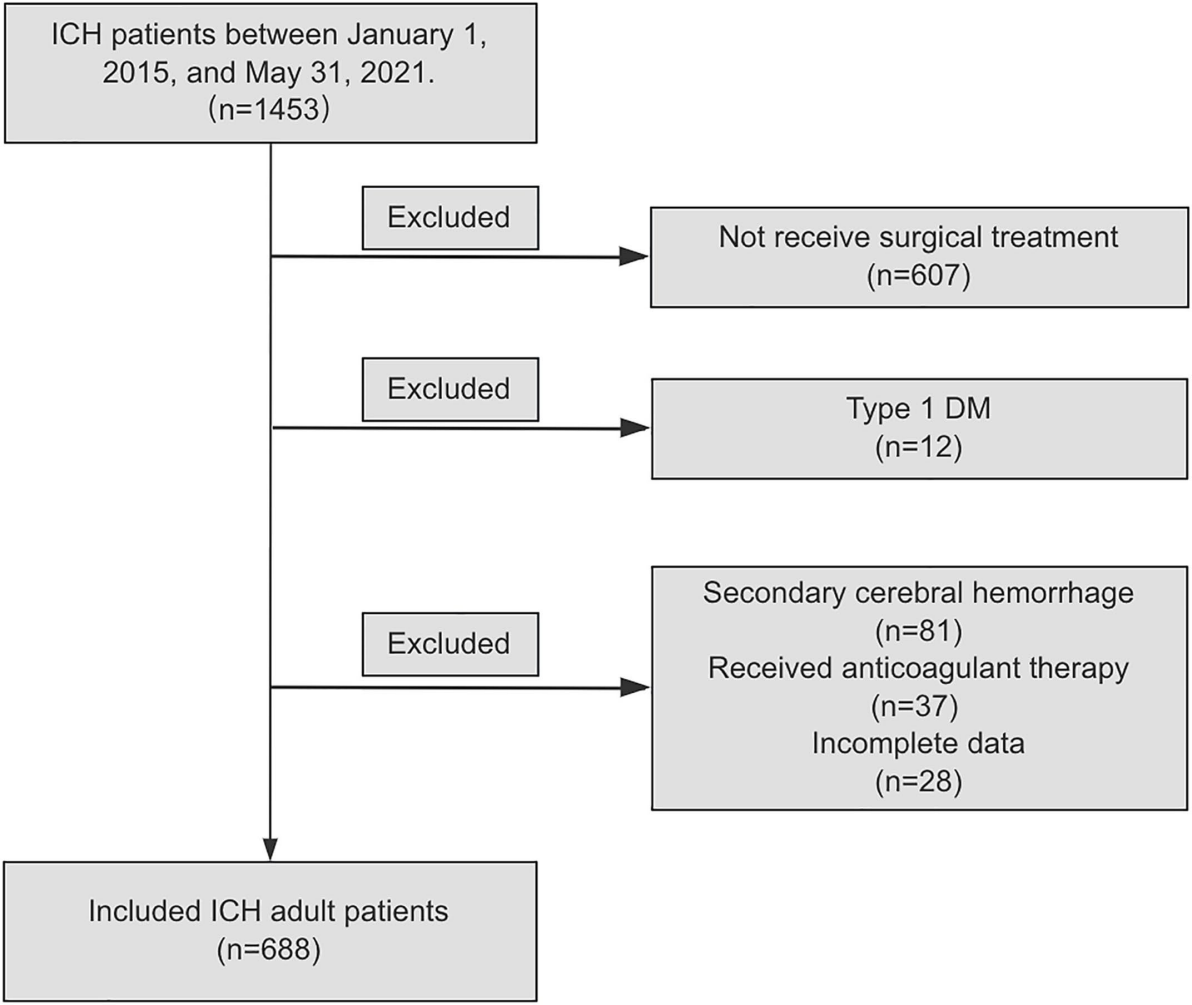
Flow diagram of study population. ICH, spontaneous intracranial hemorrhage; DM, diabetes Mellitus.

From January 1, 2015 to May 31, 2021, a total of 1,453 ICH patients who visited the neurosurgery departments of eight medical institutions, were continuously enrolled in this study. According to the inclusion and exclusion criteria, 607 (41.8%) cases were excluded because they did not receive surgical treatment. Additionally, 81 (5.6%) patients were excluded due to a diagnosis of secondary cerebral hemorrhage, including hemorrhage caused by aneurysms, cerebral and cerebrovascular malformations, moyamoya disease, and tumor stroke. 37 (2.5%) patients were excluded because they received anticoagulant therapy prior to the onset of ICH symptoms; 12 (0.8%) patients were excluded due to type 1 DM. 28 (1.9%) patients were excluded due to incomplete data. Finally, according to the inclusion and exclusion criteria, a total of 688 patients with ICH were included in the analysis, as shown in Figure 1.

### 3.2 Baseline characteristics

According to whether they were diagnosed with type 2 DM after admission, the enrolled population was divided into the no diabetes mellitus (nDM) group and the DM group. A total of 576 (83.7%) ICH patients were included in the nDM group, while 112 (16.3%) ICH patients were included in the DM group. The baseline characteristics of the two groups were shown in Table 1. As shown, significant differences were observed in age (*p* = 0.000) and sex (p = 0.000) between the two groups. Specifically, the proportion of females was larger in the DM group (38.4% VS. 22.0%), and the mean age [62.0 ± 10.2 VS. 52.1 ± 12.9] was significantly older in the DM group. Regarding living habits, the proportion of alcohol (17.9% VS. 29.2%) consumption in the DM group was lower, which may be related to the higher proportion of females in the DM group. In terms of disease history: the proportion of patients with coronary heart disease (*p* = 0.002), dyslipidemia (p = 0.000) and receiving antiplatelet therapy (*p* = 0.001) were significantly higher in the DM group compared to the nDM population. As shown in Table 1, the proportion of ventricular hematoma (p = 0.002) in the DM group was higher than that in the nDM group, but no significant statistical difference was observed in terms of hematoma volume between the two groups. Additionally, no statistical differences were observed between the two groups regarding admission systolic blood pressure, hematoma location, surgical methods, coagulation function and admission GCS. Notably, a significant statistical difference was observed in admission blood glucose levels [11.9 (9.6, 13.3) VS. 7.6 (6.6, 8.7), *p* = 0.000] between the two groups, as shown in Table 1.

**Table 1:** Patient’s baseline characteristics according to DM.

Variables	nDM (*N* = 576, 83.7%)	DM (*N* = 112, 16.3%)	*p*-value
Demographic characteristics			
Sex			
Male	449 (78.0%)	69 (61.6%)	0.000
Female	127 (22.0%)	43 (38.4%)	
Age (years)	52.1 ± 12.9	62.0 ± 10.2	0.000
Vascular risk factors			
Smoking	137 (23.8%)	21 (18.8%)	0.246
Alcohol	168 (29.2%)	20 (17.9%)	0.014
BMI	26.5 (24.3, 28.2)	26.5 (23.4, 28.6)	0.748
Admission systolic blood pressure	161.8 (143.0, 178.0)	156.7 (134.0, 170.0)	0.241
Medical history			
Hypertension	489 (84.9%)	96 (85.7%)	0.824
Coronary heart disease	35 (6.1%)	16 (14.3%)	0.002
Dyslipidemia	33 (5.7%)	25 (22.3%)	0.000
Ischemic stroke history	89 (15.5%)	23 (20.5%)	0.182
Cerebral hemorrhage history	17 (3.0%)	5 (4.5%)	0.405
Antiplatelet therapy	123 (21.4%)	40 (35.7%)	0.001
Imaging			
Side			
Left	275 (47.7%)	50 (44.6%)	0.605
Right	301 (52.3%)	62 (55.4%)	
Localization			
Lobar	130 (22.6%)	26 (23.2%)	0.902
Deep	446 (77.4%)	86 (76.8%)	
Hematoma volume (mL)	54.8 (34.7, 69.8)	55.5 (32.5, 68.2)	0.597
Ventricular hematoma	262 (45.5%)	69 (61.6%)	0.002
Surgery			
Craniotomy	256 (44.4%)	48 (42.9%)	0.215
Endoscopic surgery	45 (7.8%)	4 (3.6%)	
Minimally invasive surgery	275 (47.7%)	60 (53.6%)	
Blood transfusion	16 (4.0%)	3 (3.8%)	0.962
Laboratory test			
Platelet count 10ˆ9/L			
<125	40 (6.9%)	6 (5.4%)	0.401
125–350	520 (90.3%)	105 (93.8%)	
>350	16 (2.8%)	1 (0.9%)	
International normalized ratio	1.02 (0.95, 1.06)	1.02 (0.97, 1.08)	0.094
Glycaemia	7.6 (6.6, 8.7)	11.9 (9.6, 13.3)	0.000
Admission GCS	9.7 (7, 12)	9.8 (8, 13)	0.289

DM, diabetes mellitus; nDM, no diabetes mellitus; N, number; BMI, body mass index; GCS, Glasgow Coma Scale.

### 3.3 Differences in outcomes between groups

A total of 32 (28.6%) patients in the DM group experienced post-MACEs, including 16 (14.3%) cases of ischemic post-MACEs and 26 (23.2%) cases of hemorrhagic post-MACEs. In the nDM group, 97 (16.8%) patients experienced post-MACEs, including 45 (7.8%) cases of ischemic post-MACEs and 67 (11.6%) cases of hemorrhagic post-MACEs (Table 2). As shown in Table 2, statistically significant differences were observed between the two groups in terms of overall post-MACEs (*p* = 0.004), ischemic post-MACEs (*p* = 0.027) and hemorrhagic post-MACEs (*p* = 0.001).

**Table 2:** The association between DM and outcomes.

Outcomes	nDM (*N* = 576, 83.7%)	DM (*N* = 112, 16.3%)	*p*-value
Post-MACEs	97 (16.8%)	32 (28.6%)	0.004
Ischemic post-MACEs	45 (7.8%)	16 (14.3%)	0.027
Hemorrhagic post-MACEs	67 (11.6%)	26 (23.2%)	0.001
30-days GCS	13 (10, 15)	11.5 (8, 14)	0.003
30-days mortality	23 (4.0%)	11 (9.8%)	0.009

DM, diabetes mellitus; nDM, no diabetes mellitus; N, number; Post-MACEs, postoperative major adverse cerebrovascular events; GCS, Glasgow Coma Scale.

**Figure 2:**
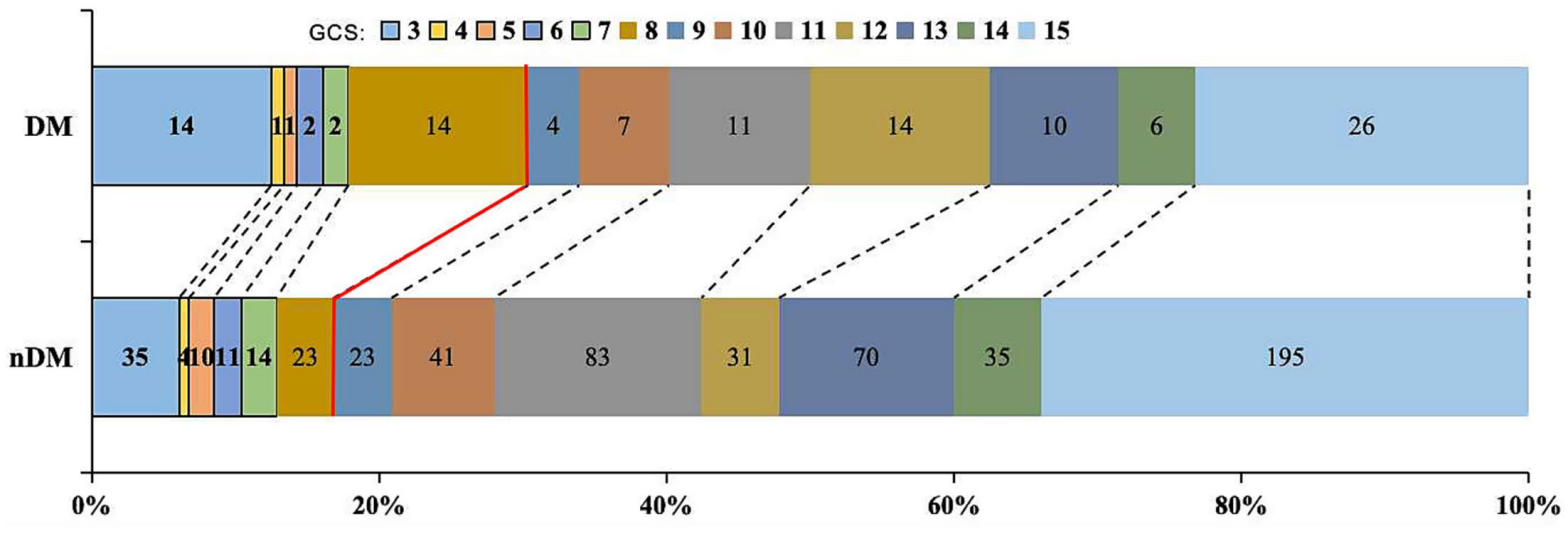
Distribution of 30-days GCS. Proportion with GCS ≤ 8: DM group 30.4% vs. nDM group 16.8%. DM, diabetes Mellitus; GCS: Glasgow Coma Scale.

Researchers further compared the 30-days GCS scores and 30-days mortality between the two groups. The results indicated that patients in the DM group had a lower 30-days GCS score [11.5 (8, 14) VS. 13 (10, 15), *p* = 0.003] and a higher 30-days mortality (9.8% VS. 4.0%, *p* = 0.009), as shown in Table 2 and Figure 2.

### 3.4 Effect of DM on outcomes

To evaluate the impact of DM on overall post-MACEs and 30-days mortality, we incorporated the confounding factors with *p* < 0.05 (as shown in Table 1) into the multivariate adjustment. Given the strong correlation between DM and blood glucose levels, blood glucose was not included in the multivariate analysis to avoid multicollinearity.

As shown in Table 3, after adjusting for sex, age, alcohol, coronary heart disease, dyslipidemia, antiplatelet therapy, and ventricular hemorrhage, DM was significantly associated with the occurrence of overall post-MACEs (1.790, 95%CI: 1.072, 2.990, *p* = 0.026) in patients with ICH. Similarly, after adjustment for the aforementioned factors, DM was significantly associated with 30-days mortality (3.593, 95%CI: 1.536, 8.406, *p* = 0.003) of patients with ICH. Additionally, our analysis revealed that coronary heart disease also serves as a risk factor for overall post-MACEs in ICH patients.

**Table 3:** The effect of DM on outcomes after adjustment.

Variables	Overall post-MACEs		30-days mortality	
	Odds ratio (95% CI)	*p*-value	Odds ratio (95% CI)	*p*-value
DM	1.790 (1.072, 2.990)	0.026	3.593 (1.536, 8.406)	0.003
Sex	0.940 (0.577, 1.531)	0.803	1.311 (0.539, 3.184)	0.550
Age	0.990 (0.973, 1.007)	0.243	0.974 (0.945, 1.004)	0.095
Alcohol	1.188 (0.750, 1.881)	0.463	0.610 (0.238, 1.566)	0.304
Coronary heart disease	1.969 (1.009, 3.842)	0.047	1.112 (0.282, 4.381)	0.879
Dyslipidemia	1.341 (0.694, 2.258)	0.383	0.254 (0.032, 2.021)	0.195
Antiplatelet therapy	1.591 (0.984, 2.572)	0.058	1.228 (0.468, 3.220)	0.676
Ventricular hematoma	1.335 (0.894, 1.994)	0.157	1.454 (0.703, 3.008)	0.313

DM, diabetes mellitus; Post-MACEs, postoperative major adverse cerebrovascular events; CI, confidence interval.

To further assess the impact of DM on outcomes, a stratified study was conducted based on the hematoma location. As shown in Supplementary Table 1, the same results were observed in deep hematomas, but not found in lobar hematomas.

### 3.5 Post-MACEs stratification study

To further assess the impact of DM on post-MACEs, a stratified analysis was conducted based on the specific types of post-MACEs, which were classified into ischemic post-MACEs and hemorrhagic post-MACEs.

**Figure 3:**
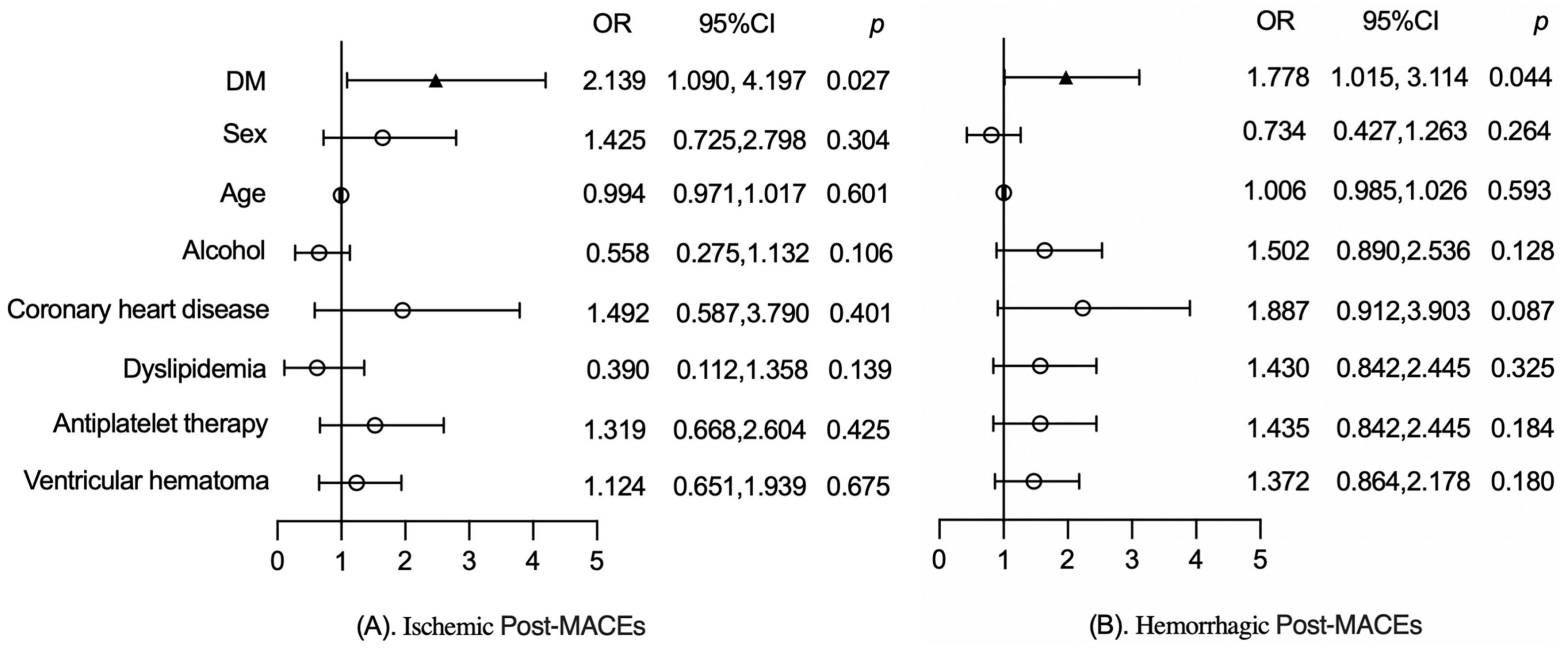
**(A)** Effect of DM on ischemic post-MACEs. **(B)** Effect of DM on hemorrhagic post-MACEs.

As shown in Figure 3A, after adjusting for factors such as sex, age, alcohol, coronary heart disease, dyslipidemia, antiplatelet therapy and ventricular hemorrhage, DM was significantly associated with ischemic post-MACEs (2.139, 95%CI:1.090, 4.197, *p* = 0.027) in patients with ICH. Similarly, adjustment for the aforementioned factors, DM was significantly associated with the hemorrhagic post-MACEs (1.778, 95%CI: 1.015, 3.114, *p* = 0.044) of ICH patients, as shown in Figure 3B.

## 4 Discussion

To evaluate the impact of DM on post-MACEs in patients with ICH, we conducted this retrospective cohort study. As demonstrated here, DM was identified not only as a risk factor for overall post-MACEs, but also as an independent risk factor for ischemic post-MACEs and hemorrhagic post-MACEs, respectively. Additionally, our analysis confirmed that DM is an independent risk factor for 30-days mortality in ICH patients.

The correlation between DM and ischemic post-MACEs has consistently drawn the attention of clinical researchers. Spiotta et al. (2011) reported that the patients with DM appeared to be more susceptible to post-MACEs during a retrospective analysis of interventional treatment for wide-neck aneurysms. Similarly, clinical studies on unruptured middle cerebral artery aneurysm clipping surgery have also observed this association (Lee et al., 2021). In 2021, a study on acute coronary syndrome revealed that type 2 DM increases the risk of MACEs, regardless of insulin use (Cavallari et al., 2021). Another retrospective study by Pantelis Stavrinou et al., which reviewed 218 carotid endarterectomy procedures, found that patients with DM had approximately 2.3 times higher risk (95% CI: 1.13–10.09) of experiencing ischemic post-MACEs within 30-days after surgery compared to nDM patients (Stavrinou et al., 2016). These findings align with our results, where we identified that the risk of ischemic post-MACEs in DM patients was approximately 2.14 times higher (95% CI: 1.090–4.197) than in nDM patients. Although the aforementioned studies (Cavallari et al., 2021; Spiotta et al., 2011; Lee et al., 2021; Stavrinou et al., 2016) primarily focused on cardiovascular and cerebrovascular populations rather than ICH patients, pathological evidence suggests that ICH is often caused by intracranial small vessel disease (Qureshi et al., 2009). Thus, the conclusions remain consistent.

The exact effect of DM on hemorrhagic post-MACEs remains controversial. Indeed, clinical evidence regarding the impact of DM on hemorrhagic post-MACEs in ICH patients is extremely limited. Cavallari et al. (2021) evaluated the influence of DM on hemorrhagic post-MACEs in patients with acute coronary syndrome. Their results indicated that only DM treated with insulin was a risk factor for hemorrhagic post-MACEs (Cavallari et al., 2021). This may be attributed to the elevated blood glucose levels observed in DM patients receiving insulin therapy, as compared to those not undergoing such treatment (Antar et al., 2023; Cavallari et al., 2021). Conversely, Hao et al. (2019) conducted a meta-analysis of 42 studies involving 10,001 cases of mechanical thrombectomy. The findings revealed that DM is a risk factor for all forms of post-MACEs (Hao et al., 2019). Similarly, Zhang et al. (2022) analyzed patients undergoing mechanical thrombectomy and found that hyperglycemia increased the occurrence of post-MACEs. These results are consistent with our study findings. Moreover, similar conclusions have also been observed in populations without cardiovascular or cerebrovascular diseases. For instance, it has been reported that DM increases postoperative bleeding complications after renal puncture (Hasegawa et al., 2022) and vitreoretinal surgery (Lauermann et al., 2021). Interestingly, these areas are also susceptible to DM (Antar et al., 2023; Demir et al., 2021; Yu et al., 2024; Cole and Florez, 2020). Therefore, it is reasonable to speculate that the influence of DM on hemorrhagic post-MACEs may be associated with small vessel injury caused by DM.

In our study, we also found that DM significantly impacts the 30-days mortality of ICH patients. Similarly, Boulanger et al. (2016) conducted a meta-analysis of 18 cohort studies involving 813 DM patients and 3,714 nDM patients. The results indicated that DM was associated with a higher 30-days mortality (1.52, 95%CI 1.28–1.81) (Boulanger et al., 2016). Subsequently, a retrospective cohort study conducted in Scotland between 2004 and 2013 among individuals aged 40 to 89 years further confirmed that both type 1 DM and type 2 DM increased the in-hospital mortality rate of ICH patients (Boulanger et al., 2017). Forti et al. (2020) categorized ICH patients into Pre-DM, DM, and nDM groups and investigated their 30-days mortality. The results showed that both Pre-DM and DM significantly elevated the 30-days mortality. However, Liebkind et al. (2018) did not observe a significant impact of DM on 30-days mortality but instead noted that DM significantly increased the one-year mortality of ICH patients. Similarly, no independent association between DM and 30-days mortality was observed in studies by Nilsson et al. (2002) and Lee et al. (2010). This discrepancy may be related to stress-induced hyperglycemia in ICH patients (Liang et al., 2024). As reported in a multicenter prospective study, stress-induced hyperglycemia was a stronger predictor of 30-days and 90-days mortality in ICH patients, compared with DM (Chen et al., 2023). Hypoglycemic treatment for DM has been shown to improve the prognosis of ICH patients (Chiu et al., 2025; Proietti et al., 2023), supporting this hypothesis. Therefore, researchers recommend implementing effective blood glucose management during the perioperative period of ICH to reduce the occurrence of post-MACEs and mortality.

With the aging of society, the impact of gender and age on disease prognosis has increasingly drawn the attention of clinical researchers. A study indicates that female with DM face a 27% higher relative risk of stroke compared to male, and they also have a higher mortality rate at age of 40 (Kautzky-Willer et al., 2016). These findings suggest that gender and age differences may amplify the effects of DM on study outcomes. However, in our multivariate analysis, gender and age did not demonstrate a statistically significant association with post-MACEs and 30-days mortality. This implies that in the studied population, gender and age did not significantly increase the occurrence rates of post-MACEs and 30-days mortality. In addition, the severity of DM seems to also have an impact on the research results. It is reported that among patients with acute coronary syndrome, the odds of developing bleeding complications in DM patients receiving insulin treatment is 2.3 times that of nDM patients (Cavallari et al., 2021); however, no statistical difference was observed between DM patients not receiving insulin treatment and nDM patients (Cavallari et al., 2021). This might be related to the fact that DM patients receiving insulin treatment have higher blood sugar levels. Manosroi et al. (2023) in a prospective study analyzed the relationship between glycated hemoglobin (HbA1c) variations and the risk of cardiovascular events in the pre-DM and DM patient populations; the results show that the highest quartile of HbA1c variability showed a statistically significant association with cardiovascular events (Manosroi et al., 2023). These studies all suggest that the blood glucose level and stability of DM can have an impact on post-MACEs (Cavallari et al., 2021; Manosroi et al., 2023). However, due to some reasons, we did not conduct further stratified studies on the blood glucose levels, HbA1c levels and insulin use. Therefore, more clinical evidence and deeper research are still needed for further exploration.

To explore the potential mechanisms underlying the effects of DM on post-MACEs, Terenzi et al. (2019) developed a high-throughput flow cytometry-based assay to quantify circulating proangiogenic and proinflammatory cell content in the peripheral blood of individuals with type 2 DM. Consistently, they detected an increased inflammatory cell burden and decreased pro-vascular progenitor content in individuals with type 2 DM, which may lead to an increase in microvascular fragility and endothelial dysfunction (Terenzi et al., 2019). Subsequently, Gómez-de Frutos et al. (2024) confirmed through preclinical studies that DM can increase cerebral edema by affecting cerebral vascularization in mice. In addition, some researchers has also confirmed that neuroinflammation, oxidative emergency stress and increased glial cell activation levels are associated with cognitive dysfunction in ICH mice models (Antar et al., 2023; Bahadar and Shah, 2021; Bahader et al., 2021). Nevertheless, the precise mechanism by which DM influences post-MACEs in ICH patients remains unclear, and further extensive studies are warranted.

As a multicenter retrospective study, our research mitigated some biases inherent in single-center data. Additionally, we assessed the impact of DM on post-MACEs within the ICH population and further stratified the analysis by the type of post-MACEs, providing valuable clinical insights for practice. However, this study has certain limitations. First, as a retrospective rather than prospective study, it has some inherent biases, such as different clinical and procedural experience among different centers, different surgical details, and lack of long-term follow-up data. This makes it challenging to adequately control for case heterogeneity during study implementation. Second, our sample size was relatively small, which may reduce the reliability of the study results. Third, we did not systematically document the pre-ICH treatment methods for DM, which may have influenced the study results. To address these shortcomings, we plan to conduct a prospective study to comprehensively evaluate the impact of DM on post-MACEs in ICH patients.

## 5 Conclusion

In conclusion, this study demonstrates that DM serves as a risk factor for ischemic post-MACEs, hemorrhagic post-MACEs, and 30-days mortality in patients with ICH.

## Data Availability

The original contributions presented in the study are included in the article/Supplementary material, further inquiries can be directed to the corresponding author.
