# Supplementary material for: Risk of postoperative major adverse cerebrovascular events in patients with spontaneous intracranial hematoma stratified by type 2 diabetes mellitus
Source: Front Hum Neurosci. 2025 Sep 11;19:1654763. doi: 10.3389/fnhum.2025.1654763 (PMC12460399; doi:10.3389/fnhum.2025.1654763)
Supplement: Supplementary file 1 [file Table_1.DOCX]

Supplementary Material

Supplementary Table 1. The effect of DM on outcomes stratified by hematoma location.

| Location | Overall post-MACEs | | 30-days mortality | |
| --- | --- | --- | --- | --- |
|  | Odds ratio (95% CI) | P-value | Odds ratio (95% CI) | P-value |
| Lobar  DM  Sex  Age  Alcohol  Coronary heart disease  Dyslipidemia  Antiplatelet therapy  Ventricular hematoma | 0.169 (0.018, 1.542)  0.263 (0.079, 0.867)  1.014 (0.964, 1.066)  1.060 (0.241, 4.652)  1.032 (0.145, 7.370)  3.900 (0.491, 30.975)  0.703 (0.161, 3.074)  0.901 (0.265, 3.059) | 0.115  0.028  0.599  0.939  0.975  0.198  0.640  0.867 | 0.840 (0.059, 12.051)  0.453 (0.071, 2.897)  0.990 (0.923, 1.062)  4.852 (0.846, 27.840)  -  -  -  7.958 (1.172, 54.047) | 0.898  0.403  0.787  0.076  0.998  0.999  0.997  0.034 |
| Deep  DM  Sex  Age  Alcohol  Coronary heart disease  Dyslipidemia  Antiplatelet therapy  Ventricular hematoma | 3.372 (1.854, 6.133)  1.804 (0.967, 3.362)  0.986 (0.967, 1.006)  1.406 (0.849, 2.330)  4.820 (2.032, 11.433)  0.641 (0.291, 1.410)  1.962 (1.131, 3.404)  1.604 (1.013, 2.539) | 0.000  0.063  0.170  0.186  0.000  0.269  0.016  0.044 | 5.550 (2.080, 14.807)  2.344 (0.769, 7.147)  0.983 (0.949, 1.018)  0.254 (0.057, 1.132)  2.115 (0.484, 9.254)  0.160 (0.018, 1.392)  1.962 (0.690, 5.584)  1.007 (0.425, 2.388) | 0.001  0.134  0.340  0.072  0.320  0.097  0.206  0.988 |

DM, diabetes mellitus; Post-MACEs, postoperative major adverse cerebrovascular events; CI, confidence interval.
